# *LIPG* SNPs, their haplotypes and gene-environment interactions on serum lipid levels

**DOI:** 10.1186/s12944-018-0942-y

**Published:** 2019-01-08

**Authors:** Shuo Yang, Rui-Xing Yin, Liu Miao, Yong-Gang Zhou, Jie Wu, Qing-Hui Zhang

**Affiliations:** Department of Cardiology, Institute of Cardiovascular Diseases, the First Affiliated Hospital, Guangxi Medical University, 22 Shuangyong Road, Nanning, 530021 Guangxi People’s Republic of China

**Keywords:** Endothelial lipase, Single nucleotide polymorphism, Haplotype, Lipids, Environmental factor

## Abstract

**Background:**

Maonan nationality is a relatively conservative and isolated minority in the Southwest of China. Little is known about the association of endothelial lipase gene (*LIPG)* single nucleotide polymorphisms (SNPs) and serum lipid levels in the Chinese populations.

**Methods:**

A total of 1280 subjects of Maonan nationality and 1218 participants of Han nationality were randomly selected from our previous stratified randomized samples. Genotypes of the four *LIPG* SNPs were determined by polymerase chain reaction-restriction fragment length polymorphism, and then confirmed by direct sequencing.

**Results:**

Several SNPs were associated with high-density lipoprotein cholesterol (rs3813082, rs2000813 and rs2097055) in the both ethnic groups; total cholesterol and apolipoprotein (Apo) A1 (rs2000813) in Han nationality; and low-density lipoprotein cholesterol, ApoB, triglyceride (rs2097055) and ApoA1 (rs3819166) in Maonan minority (*P* < 0.0125 for all after Bonferroni correction). The commonest haplotype was rs3813082T-rs2000813C-rs2097055T-rs3819166A (Han, 44.2% and Maonan, 48.7%). The frequencies of the T-C-T-A, T-C-T-G, T-T-C-G and G-T-C-G haplotypes were different between the Maonan and Han populations (*P* < 0.05–0.001). The associations between haplotypes and dyslipidemia were also different in the Han and/or Maonan populations (*P* < 0.05–0.001).

**Conclusions:**

The differences in serum lipid profiles between the two ethnic groups might partly be attributed to these *LIPG* SNPs, their haplotypes and gene-environmental interactions.

**Trial registration:**

Retrospectively registered.

## Background

Cardiovascular disease (CVD) is a major cause of morbidity and mortality in both developed and developing countries in the world [1]. Although the mortality for this condition has gradually declined over the last decades in western countries [2], it is still the second leading cause of death in China [3, 4]. Epidemiological evidence from large prospective studies has identified strong independent relations between serum lipid metabolism dysfunction and incidence of CVD [5–8]. As one of most important atherosclerosis risk factors, dyslipidemia is considered as a complicated disease caused by both environmental and genetic factors including common and rare variants in multiple genes [9, 10]. Family history and twin studies have showed that genetic polymorphisms can determine 40–60% of the interindividual variation in plasma lipid phenotypes [9]. Therefore, human genetic studies can improve understanding of lipid-related loci and provide potential new targets for future individual therapy.

Human endothelial lipase gene (*LIPG*, also as known as EL, EDL, PRO719; Gene ID: 9388; HGNC ID: 6623), which maps to chromosome 18q12.1–q12.3, spans 30 kb with 10 exons and encodes a polypeptide of 500 amino acids. *LIPG* belongs to the triglyceride (TG) lipase gene family with 46% identity to lipase member H (LIPH), 45% to lipoprotein lipase (LPL), 40% to hepatic lipase (HL) and 31% to pancreatic lipase (PNLIP) [11–13]. Research shows that *LIPG* is synthesized not only by endothelial cells, but also by other types of cells such as macrophages and hepatocytes [14]. According to the previous studies, *LIPG* is the key enzyme to regulate and hydrolyzes serum high-density lipoprotein (HDL) [15], to generate free fatty acids and low-lipid apolipoprotein (Apo) A1 [16]. In animal experiments, mouse models of over expressing *LIPG* have reduced high-density lipoprotein cholesterol (HDL-C) levels and those of deficient *LIPG* have marked elevation of HDL-C levels [17, 18]. Another recent study has demonstrated that *LIPG* expression in mice may impact other atherogenic lipoproteins, including low-density lipoprotein cholesterol (LDL-C) and ApoB [19]. Prior genome-wide association studies (GWASes) of *LIPG* variants in human populations have generally shown an association of *LIPG* rs2000813 SNP with increased HDL-C levels and also play an important role in development of CVD [20–22], although some studies have contradictory conclusions [23]. Several previous studies also found that rs3813082 polymorphism in the *LIPG* promoter was associated with plasma HDL-C levels [24, 25]. In 2011, a study identified that the *LIPG* rs3819166 SNP had some effect on HDL-C level in Caucasian, however, the exact mechanism of this variant has not been established [26]. As compared to the rs2000813 polymorphism, the rs2097055 SNP has been studied less. Vergeer et al had taken several SNPs into consideration that including *LIPG* rs2000813, rs6507931 and rs2097055, only found that a modest association between the rs2000813 SNP and parameters of HDL-C metabolism, but the rs2097055 variant was associated with deep venous thrombosis (DVT) risk [27]. These conflicting results may be due to differences in the ethnic composition of the samples or in serum lipid levels of the study populations.

Among 56 nationalities in China, Maonan nationality is an isolated minority with a population of 107,166 according to the sixth national census statistics of China in 2010. Approximately 80% of the total Maonan people reside in the Huanjiang Maonan Autonomous County in the Northwestern of Guangxi Zhuang Autonomous Region. Maonan people have their own national language and special customs and culture, including their traditional dress, intra-ethnic marriages, unique dietary habits and lifestyle which are different from those of local Han ethnic group [28]. They mainly engaged in agriculture and were good at raising beef cattle and prepare the bamboo hat. Maonan people like to eat pickle sour meat, snails and animal offals which contain abundant saturated fatty acid. In a previous study, we found that the intakes of total energy, total fat and dietary cholesterol were higher in Maonan than in Han and the prevalence of hyperlipidemia was also higher in Maonan than in Han [29]. Importantly, Maonan ethnic group is a relatively conservative minority not only in nature environment but also in custom of intra-ethnic marriage. For example, more than 80% of the Maonan people share the same surname: Tan, suggesting that their genetic background may be less heterogeneous within the population. It is still dubious whether the *LIPG* SNPs are associated with serum lipid levels in this population or whether it shows ethnic-specificity. Therefore, the purpose of the current study was to examine the association of four *LIPG* SNPs (rs2000813, rs3819166, rs2097055 and rs3813082), their haplotypes and gene-environment (G × E) interactions on serum lipid traits in the Maonan and Han populations.

## Methods

### Subjects

The participants in the present study included 1280 unrelated individuals of Maonan (581 males, 45.39% and 699 females, 54.61%) and 1218 unrelated subjects of Han (571 males, 46.88% and 647 females, 53.12%). They were randomly selected from our previous stratified randomized samples. All participants were agricultural workers living in Huanjiang Maonan Autonomous County, Guangxi Zhuang Autonomous Region, People’s Republic of China. The age of the participants ranged from 25 to 80 years, with a mean age of 56.0 ± 11.6 years in Han and 57.1 ± 14.0 years in Maonan (*P* > 0.05), respectively. All study subjects were essentially healthy with no history of CVD such as coronary artery disease (CAD) and stroke, diabetes, hyper- or hypo-thyroids, and chronic renal disease. They were free from medications known to affect serum lipid levels. The investigations were carried out following the rules of the Declaration of Helsinki of 1975 (http://www.wma.net/en/30publications/10policies/b3/), revised in 2008. We excluded the subjects who had a history of taking medications known to affect serum lipid levels (lipid-lowering drugs such as statins or fibrates, beta blockers, diuretics, or hormones) before the blood sample was drawn. The present study was approved by the Ethics Committee of the First Affiliated Hospital, Guangxi Medical University (No: Lunshen-2014-KY-Guoji-001; March 7, 2014). Informed consent was taken from all participants after they received a full explanation of the study.

### Epidemiological survey

The survey was carried out using internationally standardized methods, following a common protocol [30]. Information on demographics, socioeconomic status, and lifestyle factors was collected with standardized questionnaires. The alcohol information included questions about the number of liangs (about 50 g) of rice wine, corn wine, rum, beer, or liquor consumed during the preceding 12 months. Alcohol consumption was categorized into groups of grams of alcohol per day: ≤ 25 and >  25. Smoking status was categorized into groups of cigarettes per day: ≤ 20 and >  20. The methods of measuring blood pressure, height, weight, body mass index (BMI) and waist circumference parameters were based on the previous study [31].

### Biochemical measurements

A venous blood sample of 5 ml was obtained from the participants, after at least 12 h of fasting. A part of the sample (2 mL) was collected into glass tubes and used to determine serum lipid levels, and another part (3 mL) was shifted to tubes with anticoagulants (4.80 g/L citric acid, 14.70 g/L glucose and 13.20 g/L tri-sodium citrate) and used to extract deoxyribonucleic acid (DNA). The levels of fasting serum total cholesterol (TC), TG, HDL-C and LDL-C in the samples were performed by enzymatic methods with commercially available kits (RANDOX Laboratories Ltd., Ardmore, Diamond Road, Crumlin Co. Antrim, United Kingdom, BT29 4QY; Daiichi Pure Chemicals Co., Ltd., Tokyo, Japan). Fasting serum ApoA1 and ApoB levels were measured by the immunoturbidimetric immunoassay using a commercial kit (RANDOX Laboratories Ltd.). All determinations were performed with an auto-analyzer (Type 7170A; Hitachi Ltd., Tokyo, Japan) in the Clinical Science Experiment Center of the First Affiliated Hospital, Guangxi Medical University [32].

### Tagging SNP selection

We selected four *LIPG* SNPs with the following steps: (i) The *LIPG* SNPs were selected from previous GWASes which were associated with lipid-metabolism especially HDL-C. (ii) Tagging SNPs, which were established by Haploview (Broad Institute of MIT and Harvard, USA, version 4.2) and functional SNPs predicted to lead to serum lipid changes from current version of online resource (1000 Genome Project Database). (iii) SNP information was obtained from NCBI dbSNP Build 132 (https://www.ncbi.nlm.nih.gov/snp/). (iv) SNPs were restricted to minor allele frequency (MAF) higher than 1% in European ancestry from the Human Genome Project Database. The four SNPs were selected by the block-based approach. This strategy was enabled by the correlations between tagging SNPs as manifested as linkage disequilibrium (LD).

### DNA amplification and genotyping

Genomic DNA of the samples was extracted from peripheral blood leucocytes according to the phenol-chloroform method [33]. The extracted DNA was stored at 4 °C until analysis. Genotyping of the *LIPG* SNPs was performed by polymerase chain reaction and restriction fragment length polymorphism (PCR-RFLP). The sequences of the forward and backward primers, restriction enzymes used and the size of the restriction fragments are shown in Table 1. The PCR products of the samples (two samples of each genotype) were sequenced with an ABI Prism 3100 (Applied Biosystems, international Equipment Trading Ltd., Vernon Hill, IL, USA) in Shanghai Sangon Biological Engineering Technology & Services Co., Ltd., China.

**Table 1:** The sequences of forward and backward primers, restriction enzymes for genotyping of the *LIPG* SNPs

SNP	Primer sequence	PCR product	Restriction enzyme	Restriction fragment	Allele
rs3813082	5'-GACACCCAGATCCTCCTCTC-3' 3'-AGGAGGACAAAGGGGATGAC-5'	210 bp	*Hha*I	210 117 + 93	G T
rs2000813	5'-CATGAGCTGAGATTGTTGTCAGTGC-3' 3'-CAGTCAACCACAACTACATTGGCGTCTTTCTCTCAT-5'	254 bp	*Nde*I	254 217 + 37	C T
rs2097055	5'-TCAGGATTCTCGAGCAGTCC-3' 3'-CTTAGGGGAGGCCAAAAGGA-5'	421 bp	*Bcc*I	421 283 + 138	C T
rs3819166	5'-CCGGACGATGCAGATTTTGT-3' 3'-CATTGCACTCTAACCTGGGC-5'	473 bp	*Ssp*I	473 307 + 166	G A

### Diagnostic criteria

The normal values of serum TC, TG, HDL-C, LDL-C, ApoA1, ApoB levels and the ApoA1/ApoB ratio in our Clinical Science Experiment Center were 3.10–5.17, 0.56–1.70, 1.16–1.42, 2.70–3.10 mmol/L, 1.20–1.60, 0.80–1.05 g/L and 1.00–2.50, respectively. The individuals with TC > 5.17 mmol/L and/or TG > 1.70 mmol/L were defined as hyperlipidemic [34]. Hypertension diagnosis standard is according to the criteria of 1999 and 2003 World Health Organization-International Society of Hypertension Guidelines for the management of hypertension [35]. The diagnostic criteria of overweight and obesity were according to the Cooperative Meta-analysis Group of China Obesity Task Force. Normal weight, overweight and obesity were defined as a BMI < 24, 24–28 and > 28 kg/m^2^, respectively.

### Statistical analyses

Data analysis was performed with the statistical software package SPSS 22.0 (SPSS Inc., Chicago, Illinois). The quantitative variables were presented as mean ± standard deviation, that are normally distributed, and the medians and interquartile ranges for TG, which is not normally distributed [36]. The allele, genotype and haplotype frequencies between the two ethnic groups were analyzed by the chi-squared test; and the Hardy-Weinberg equilibrium was verified with the standard goodness-of-fit test. Haploview (Broad Institute of MIT and Harvard, USA, version 4.2) was used to analyze the haplotype frequencies and the pair-wise LD among the detected SNPs. The general characteristics between the two ethnic groups were compared by the Student’s unpaired *t*-test. The association between genotypes and serum lipid parameters was tested by covariance analysis (ANCOVA). Gender, age, BMI, blood pressure, alcohol consumption and cigarette smoking were adjusted for the statistical analysis. The genotype frequencies of the four SNPs and any SNP associated with the serum lipid profiles at the value of *P* < 0.0125 (corresponding to *P* < 0.05 after adjusting for 4 independent tests by the Bonferroni correction) were considered statistically significant. Multivariable linear regression analyses with stepwise modeling were used to determine the correlation between the genotypes (common homozygote genotype = 1, heterozygote genotype = 2, rare homozygote genotype = 3) or alleles (the minor allele non-carrier = 1, the minor allele carrier = 2) and several demographic characteristics and lifestyle factors with serum lipid levels in combined population of Maonan and Han, Maonan, Han, males and females; respectively. Two-sided *P* value of less than 0.05 was considered statistically significant.

## Results

### Demographic and clinical characteristics

The demographic and clinical characteristics between the Han and Maonan populations are summarized in Table 2. The levels of systolic blood pressure, diastolic blood pressure, pulse pressure, the percentages of subjects who consumed alcohol and the levels of serum TG were lower in Han than in Maonan (*P* < 0.05–0.001), whereas the height, weight and serum ApoA1 levels were higher in Han than in Maonan (*P* < 0.05–0.001). There were no significant differences in the gender ratio, age structure, waist circumference, the percentage of cigarette smoking, BMI, glucose, serum TC, HDL-C, LDL-C and the ApoA1/ApoB ratio between the two ethnic groups (*P* > 0.05 for all).

**Table 2:** Comparison of demographic, lifestyle characteristics and serum lipid levels between the Han and Maonan populations

Parameter	Han	Maonan	*t* (*x*^2^)	*P*
Number	1218	1280		
Male/female	571/647	581/699	0.545	0.486
Age (years)	56.0 ± 11.6	57.1 ± 14.0	1.381	0.164
Height (cm) Weight (kg)	153.79 ± 7.84 53.53 ± 9.03	152.99 ± 7.99 52.66 ± 10.52	2.461 2.149	0.014 0.032
Body mass index (kg/m^2^)	22.60 ± 3.34	22.43 ± 3.94	1.168	0.243
Waist circumference (cm)	75.39 ± 7.86	75.67 ± 9.25	−0.793	0.428
Smoking status [*n* (%)]				
Non-smoker	975(80.05)	1002(78.27)		
≤ 20 cigarettes/day	203(16.67)	248(19.40)		
> 20 cigarettes/day	40(3.28)	30(2.33)	4.751	0.096
Alcohol consumption [*n* (%)]				
Non-drinker	1026(84.20)	1014(79.27)		
≤ 25 g/day	103(8.48)	156(12.19)		
> 25 g/day	89(7.32)	110(8.59)	11.601	0.003
Systolic blood pressure (mmHg)	129.33 ± 19.69	135.66 ± 25.48	−6.678	1.64E-11
Diastolic blood pressure (mmHg)	81.36 ± 11.28	84.68 ± 13.01	−6.633	4.05E-11
Pulse pressure (mmHg)	47.97 ± 15.25	50.98 ± 18.98	−4.264	0.001
Glucose (mmol/L)	5.89 ± 1.49	5.93 ± 1.29	−0.651	0.515
Total cholesterol (mmol/L)	4.97 ± 1.07	5.04 ± 1.14	−0.800	0.424
Triglyceride (mmol/L)	1.25(0.53)	1.30(0.41)	−2.177	0.030
HDL-C (mmol/L)	1.68 ± 0.51	1.63 ± 0.39	1.279	0.201
LDL-C (mmol/L)	2.88 ± 0.89	2.90 ± 0.81	−0.120	0.905
ApoA1 (g/L)	1.37 ± 0.40	1.33 ± 0.26	−3.127	0.002
ApoB (g/L)	0.84 ± 0.17	0.87 ± 0.20	−2.878	0.091
ApoA1/ApoB	1.67 ± 0.51	1.66 ± 0.50	0.310	0.756

*HDL-C* high-density lipoprotein cholesterol, *LDL-C* low-density lipoprotein cholesterol, *Apo* apolipoprotein. The value of triglyceride was presented as median (interquartile range), the difference between the two ethnic groups was determined by the Wilcoxon-Mann-Whitney test

### Results of electrophoresis and genotyping

**Fig. 1:**
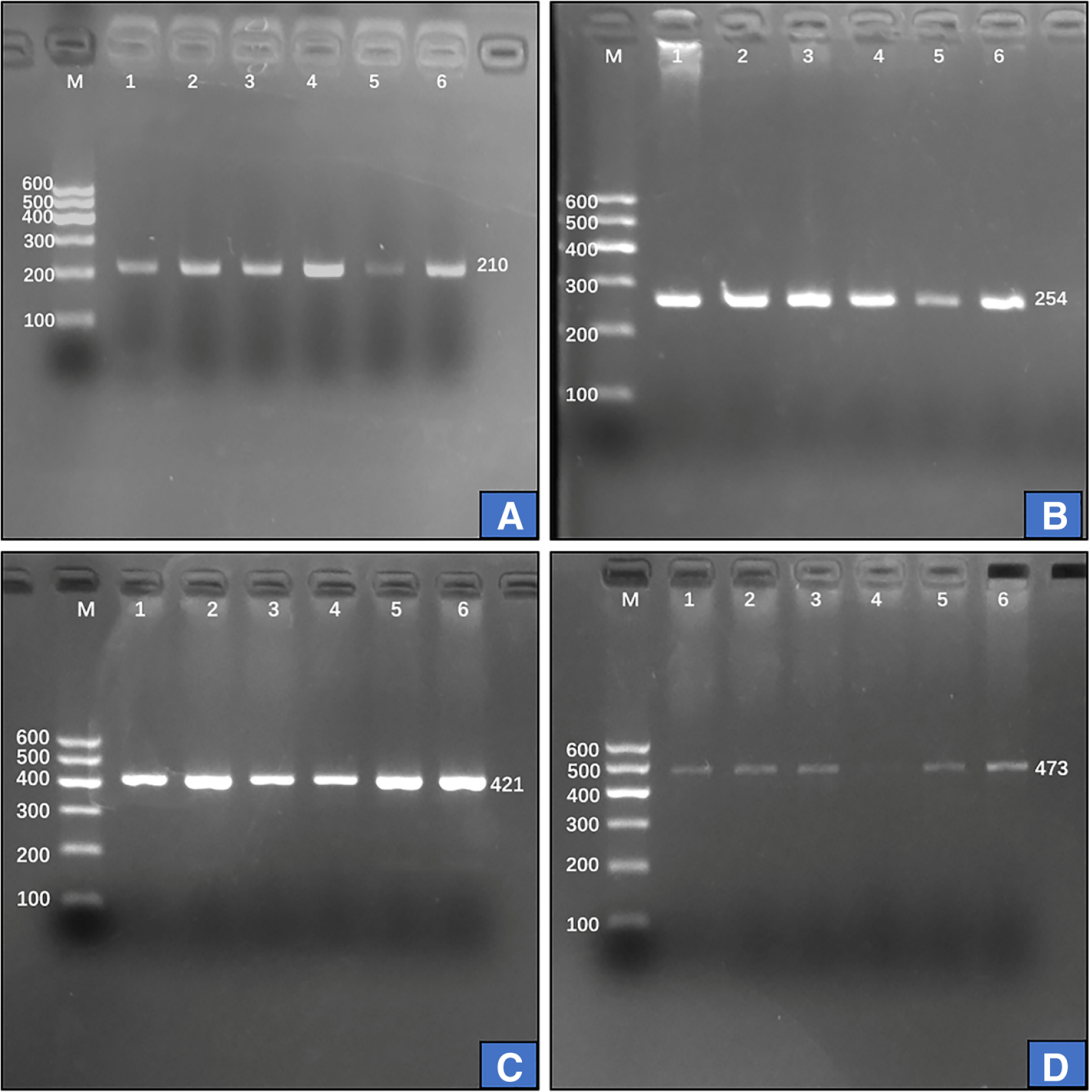
Electrophoresis of polymerase chain reaction products of the samples. Lane M is the 100 bp marker ladder; lanes 1–6 are samples; the PCR products of **a** (rs3813082), **b** (rs2000813), **c** (rs2097055) and **d** (rs3919166) were 210-, 254-, 421- and 473-bp bands; respectively

**Fig. 2:**
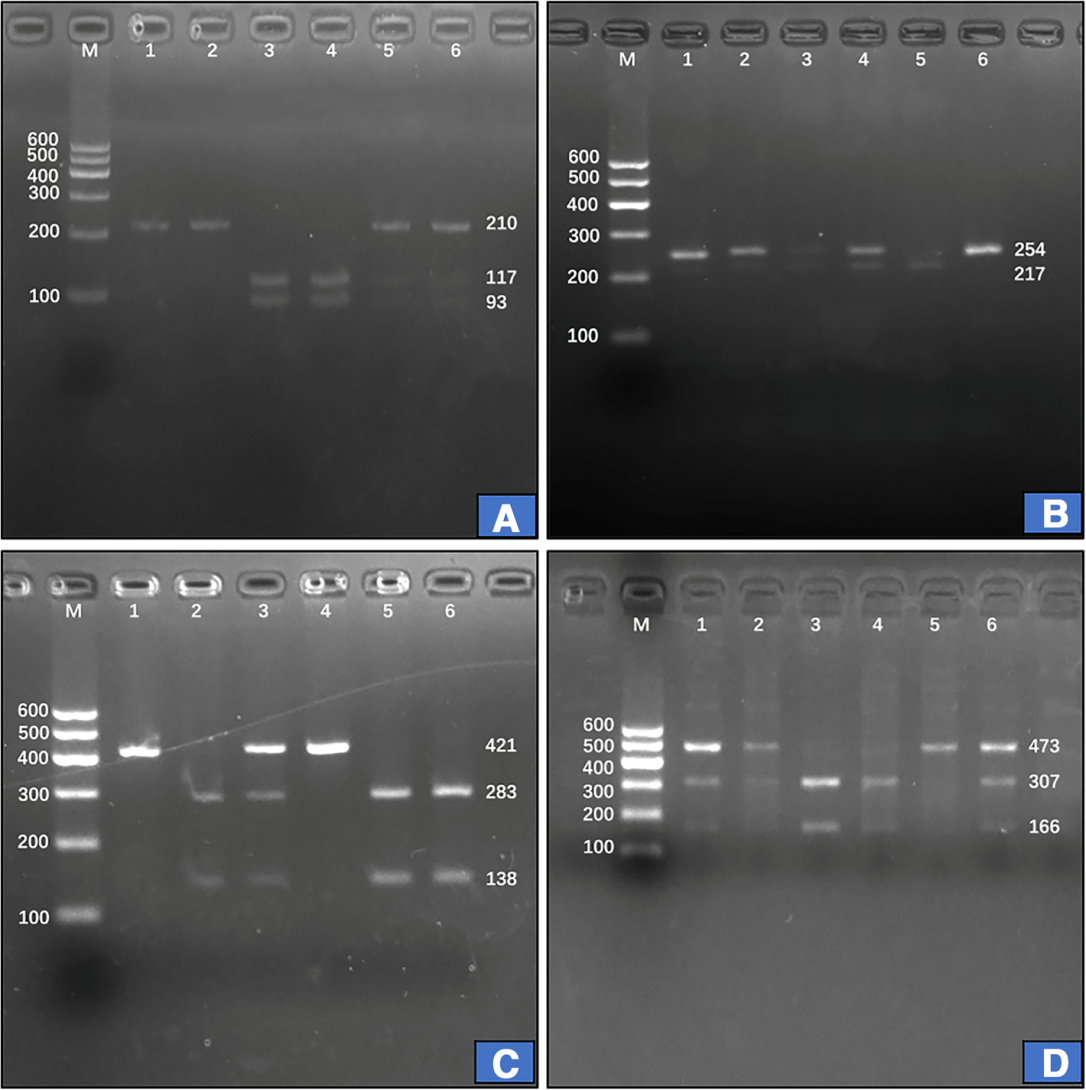
Genotyping of the four *LIPG* SNPs. Lane M is the 100 bp marker ladder; lanes 1–6 are samples. **a** (rs3813082): lanes 1 and 2, GG genotype (210 bp); lanes 3 and 4, TT genotype (117- and 93-bp); and lanes 5 and 6, GT genotype (210-, 117- and 93-bp); **b** (rs2000813): lanes 2 and 6, CC genotype (254-bp); lanes 3 and 4, CT genotype (254-, 217- and 37-bp); and lanes 1 and 5, TT genotype (254- and 217-bp); **c** (rs2097055): lanes 1 and 4, CC genotype (421-bp); lanes 2, 5 and 6, TT genotype (283- and 138-bp); and lane 3, CT genotype (421-, 283- and 138-bp); **d** (rs3819166): lanes 2 and 5, GG genotype (473-bp); lanes 3 and 4, AA genotype (307- and 166-bp); and lanes 1 and 6, AG genotype (473-, 307- and 166-bp). The bands less than 90-bp fragments were not visible in the gel owing to their fast migration speed

**Fig. 3:**
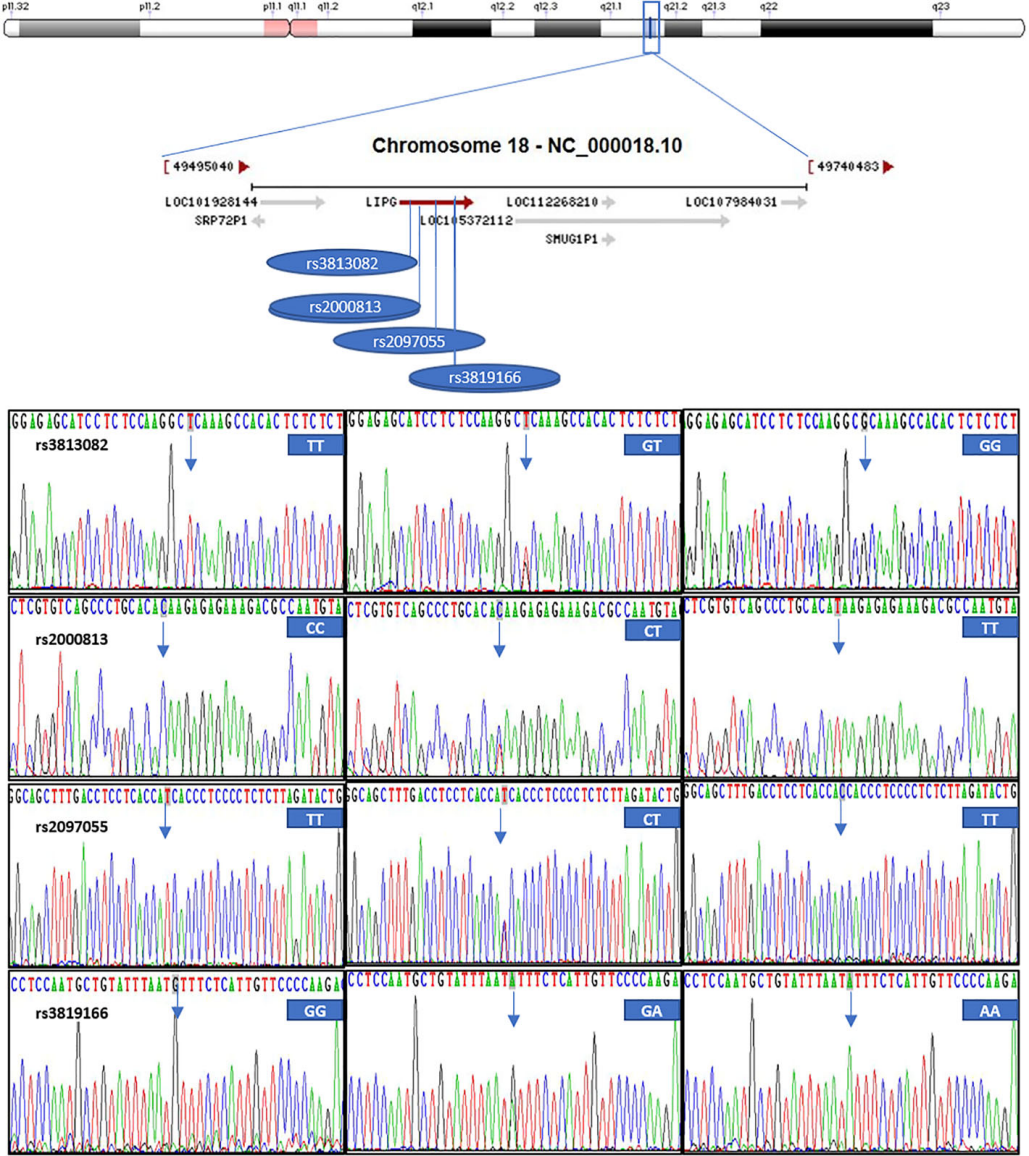
A part of the nucleotide sequences of the four *LIPG* SNPs

After the genomic DNA of the samples was amplified using PCR and visualized with 2% agarose gel electrophoresis, the PCR products of *LIPG* rs3813082, rs2000813, rs2097055 and rs3819166 SNPs were seen, they were 210-, 254-, 421- and 473-bp nucleotide sequences; respectively (Fig. 1). After restriction fragment length polymorphism (RFLP) reaction and then imaged by 2% agarose gel electrophoresis, the genotypes of the SNPs identified were labeled according to the presence and absence of the enzyme restriction sites (Fig. 2). The nucleotide direct sequencing confirmed the genotypes shown by PCR-RFLP; respectively (Fig. 3).

### Genotypic and allelic frequencies

The genotypic distribution of the four loci was in Hardy-Weinberg equilibrium (*P* > 0.05 for all). The genotypic frequencies of the *LIPG* rs3813082 SNP were significantly different between the Maonan and Han ethnic groups (*P* = 1.4E-04, *P* < 0.0125 was considered statistically significant after the Bonferroni correction); and the allelic frequencies of the four *LIPG* SNPs were significantly different between the Maonan and Han populations (*P* < 0.05–0.01; Tables 3 and 4).

**Table 3:** Genotype frequencies of 4 *LIPG* SNPs between the Han and Maonan ethnic groups [*n* (%)]

SNP	Genotype	Han (*n* = 1218)	Maonan (*n* = 1280)	*x* ^2^	*P*
rs3813082 T > G	TT	754(61.90)	889(69.45)		
	TG	397(32.59)	347(27.11)	17.691	1.14E-04
	GG	67(5.50)	44(3.44)		
	*P*_HWE_	0.119	0.163		
rs2000813 C > T	CC	639(52.46)	737(57.58)		
	CT	499(40.97)	479(37.42)	7.337	0.025
	TT	80(6.57)	64(5.00)		
	*P*_HWE_	0.159	0.219		
rs2097055 T > C	TT	437(35.88)	530(41.41)		
	CT	599(49.18)	580(45.39)	8.115	0.017
	CC	182(14.94)	170(13.28)		
	*P*_HWE_	0.322	0.589		
rs3819166 G > A	GG	381(31.28)	340(26.56)		
	GA	611(50.16)	668(52.19)	7.063	0.022
	AA	226(18.56)	272(21.25)		
	*P*_HWE_	0.467	0.089		

A value of *P* < 0.0125 (corresponding to *P* < 0.05 after adjusting for 4 independent tests by the Bonferroni correction) was considered statistically significant

**Table 4:** Allele frequencies of 4 *LIPG* SNPs between the Han and Maonan populations [*n* (%)]

SNP	Allele	Han (*n* = 1218)	Maonan (*n* = 1280)	*x* ^2^	*P*
rs3813082	T/G	1905 (78.20) / 531 (21.80)	2125 (83.01) / 435 (16.99)	18.484	1.17E-05
rs2000813	C/T	1777 (72.95) / 659 (27.05)	1953 (76.29) / 607 (23.71)	7.367	0.007
rs2097055	T/C	1473(60.47) / 963 (39.53)	1640 (64.06) / 920 (35.94)	6.867	0.009
rs3819166	G/A	1373 (56.36) / 1063(43.64)	1348 (52.66) /1212 (47.34)	6.915	0.009

### Haplotype frequencies

**Fig. 4:**
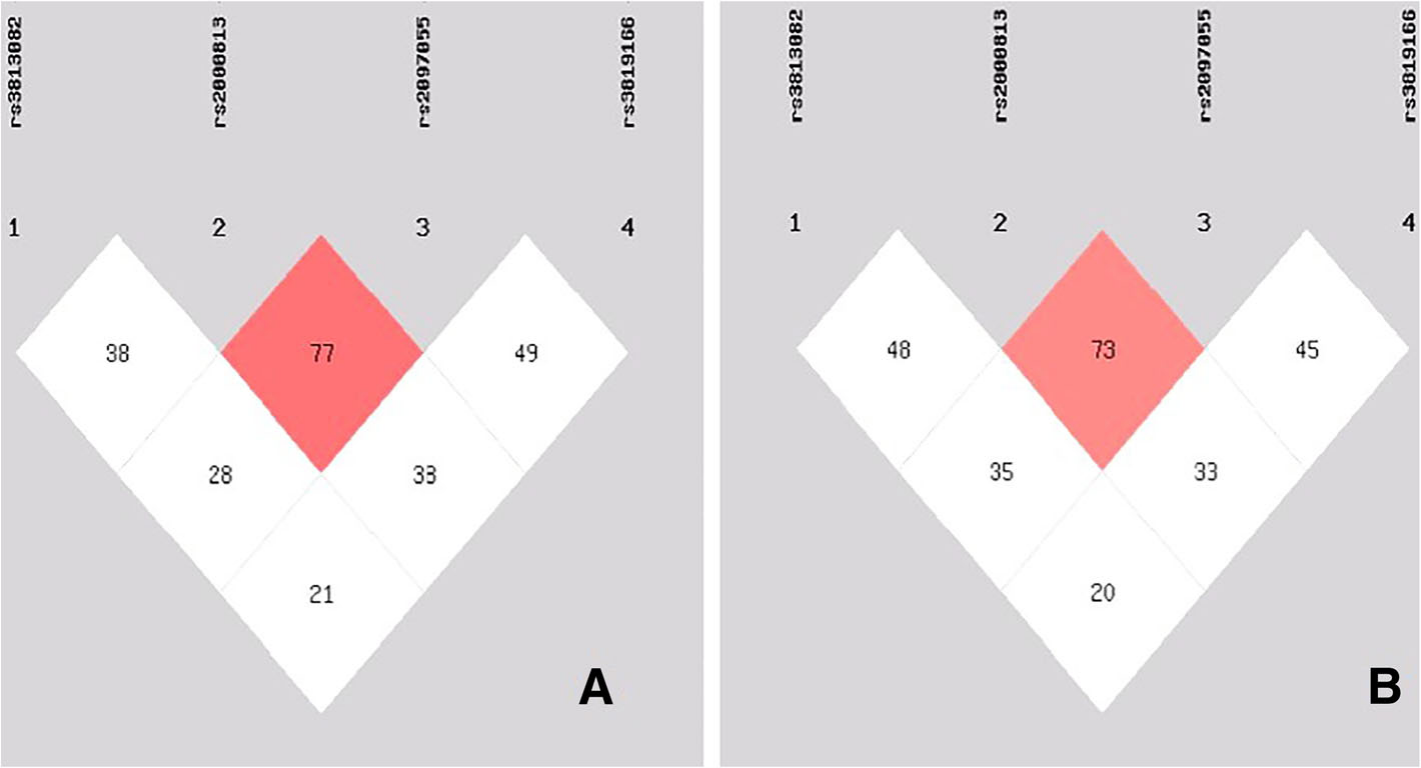
Linkage disequilibrium (LD) analyses of the four *LIPG* SNPs. LD status was shown in **a** (Han) and **b** (Maonan) among 1 (rs3813082), 2 (rs2000813), 3 (rs2097055) and 4 (rs3819166) SNPs. The LD status is expounded by the *r*^2^

A LD was noted between the four SNPs (Fig. 4). The haplotype frequencies are listed in Table 5. Six haplotypes were identified in the cluster in both populations, and the rare haplotypes (frequency < 3%) have been dropped. The commonest haplotype was rs3813082T-rs2000813C-rs2097055T-rs3819166A. The frequencies of the T-C-T-A, T-C-T-G, T-T-C-G and G-T-C-G haplotypes were different between the Maonan and Han populations (*P* < 0.05–0.001).

**Table 5:** Haplotype frequencies among 4 *LIPG* SNPs between the Han and Maonan populations [*n* (%)]

Haplotype	Han	Maonan	*x* ^2^	*P*-value	Odd Ratio [95%CI]
A-B-C-D					
T-C-T-A	1075 (0.442)	1245 (0.487)	8.021	0.004642	0.850 [0.760–0.951]
T-C-T-G	366 (0.150)	447 (0.175)	4.650	0.031103	0.847 [0.728–0.985]
T-T-C-G	323 (0.133)	256 (0.100)	14.081	0.000177	1.395 [1.172–1.661]
T-C-C-G	143 (0.059)	160 (0.063)	0.211	0.646340	0.947 [0.750–1.195]
G-T-C-G	467 (0.192)	416 (0.163)	8.420	0.003728	1.241 [1.072–1.436]
G-C-T-G	60 (0.025)	32 (0.013)	–	–	–

*A* rs3813082 T > G, *B* rs2000813 C > T, *C* rs2097055 T > C, and *D* rs3819166 G > A SNPs

### Genotypes and serum lipid levels

**Fig. 5:**
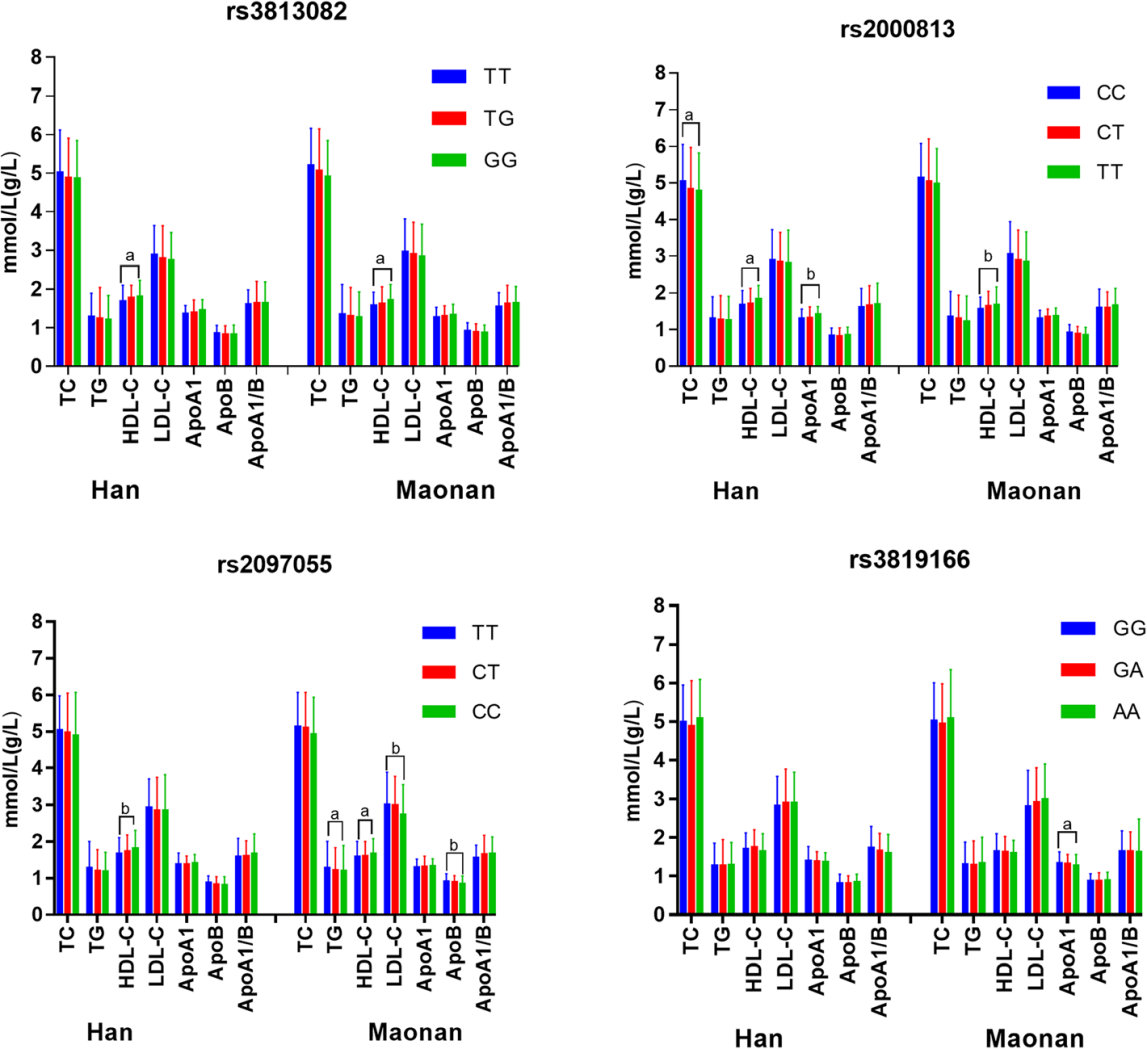
Association of single SNP and serum lipid levels. *TC* total cholesterol, *TG* triglyceride, *HDL-C* high-density lipoprotein cholesterol, *LDL-C* low-density lipoprotein cholesterol, *Apo* apolipoprotein. ^a^*P* < 0.0125 (was considered statistically significant after the Bonferroni correction) and ^b^*P* < 0.001

Figure 5 describes the association between genotypes and serum lipid concentrations. Three SNPs (rs3813082, rs2000813 and rs2097055) were associated with HDL-C in the both ethnic groups (*P* < 0.0125). The levels of ApoA1 and TC (rs2000813) in the Han population were different among the three genotypes, whereas the levels of LDL-C, ApoB and TG (rs2097055) and ApoA1 (rs3819166) were different among the genotypes in the Maonan minority.

### Haplotypes and serum lipid profiles

**Fig. 6:**
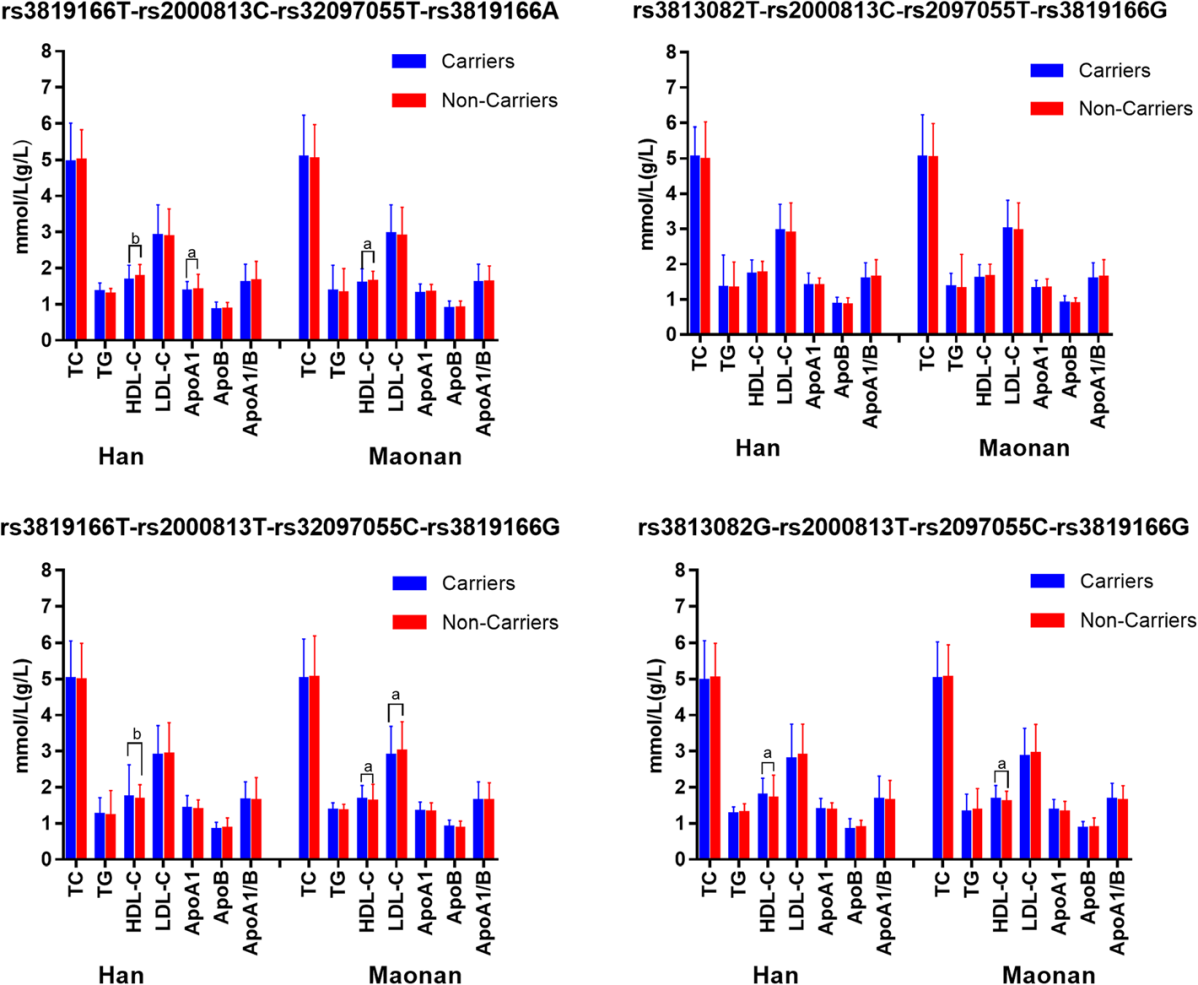
Association of the haplotypes and serum lipid traits. *TC* total cholesterol, *TG* triglyceride, *HDL-C* high-density lipoprotein cholesterol, *LDL-C* low-density lipoprotein cholesterol, *Apo* apolipoprotein. ^a^*P* < 0.0125 (was considered statistically significant after the Bonferroni correction) and ^b^*P* < 0.001

The association of the haplotypes and serum lipid profiles is shown in Fig. 6. The commonest haplotype T-C-T-A was associated with lower serum HDL-C levels in both Maonan and Han populations and lower levels of ApoA1 in Maonan population (*P <* 0.0125). The haplotype carriers of T-T-C-G and G-T-C-G in the two ethnic groups had higher HDL-C levels than the haplotype non-carriers; and the T-T-C-G carriers in the Maonan population had lower LDL-C levels than the T-T-C-G non-carriers (*P <* 0.0125). There were no differences in serum lipid parameters between the carriers and non-carriers of T-C-T-G haplotype in the two populations.

### Correlated factors for serum lipid parameters

The correlation between genotypes of four SNPs and serum lipid parameters is shown in Table 6. The *LIPG* genotypes were associated with HDL-C (rs3813082, rs2000813 and rs2097055) in both ethnic groups; ApoA1 (rs2000813) in the Han population; and TG, LDL-C (rs2097055) and ApoA1 (rs3819166) in the Maonan ethnic group. Serum lipid parameters were also correlated with several demographic characteristics and lifestyle factors such as sex, age, alcohol consumption, cigarette smoking, blood pressure, blood glucose, waist circumference, and BMI in both ethnic groups (*P* < 0.05–0.001; Table 7).

**Table 6:** Association between the *LIPG* SNPs or their haplotypes and serum lipid traits in the Han and Maonan populations

Lipid	SNP/Haplotype	Genotype/Haplotype	B	Std.error	Beta	*t*	*P*
Han							
HDL-C	rs3813082 genotype	TT/TG/GG	0.050	0.020	0.120	2.506	0.013
	rs2000813 genotype	CC/CT/TT	0.021	0.012	0.053	1.899	0.040
	rs2097055 genotype	TT/CT/CC	0.052	0.021	0.065	2.520	0.012
	T-C-T-A	carriers/non-carriers	0.085	0.030	0.090	2.864	0.004
	T-T-C-G	carriers/non-carriers	0.055	0.023	0.069	2.355	0.019
	G-T-C-G	carriers/non-carriers	−0.007	0.001	− 0.169	−5.200	0.000
ApoA1	rs2000813 genotype	CC/CT/TT	−0.010	0.002	−0.230	−4.505	0.000
Maonan							
HDL-C	rs3813082 genotype	TT/TG/GG	0.097	0.025	0.124	3.968	0.000
	rs2000813 genotype	CC/CT/TT	0.036	0.010	0.093	3.703	0.000
	rs2097055 genotype	TT/CT/CC	0.072	0.023	0.098	3.106	0.002
	T-C-T-A	carriers/non-carriers	−0.021	0.008	−0.073	−2.543	0.011
	T-T-C-G	carriers/non-carriers	−0.013	0.006	−0.062	−2.332	0.020
	G-T-C-G	carriers/non-carriers	−0.265	0.080	−0.144	−3.313	0.001
TG	rs2097055 genotype	TT/CT/CC	0.007	0.003	0.153	2.789	0.006
LDL-C	rs2097055 genotype	TT/CT/CC	−0.189	0.058	− 0.168	−3.227	0.001
	T-T-C-G	carriers/non-carriers	−0.145	0.073	0.063	1.979	0.040
ApoA1	rs3819166 genotype	GG/AG/AA	0.002	0.001	0.158	2.838	0.005

*TC* total cholesterol, *TG* triglyceride, *HDL-C* high-density lipoprotein cholesterol, *LDL-C* low-density lipoprotein cholesterol, *ApoA1* apolipoprotein A1, *ApoB* apolipoprotein B, *ApoA1/ApoB* the ratio of apolipoprotein A1 to apolipoprotein B; *B* unstandardized coefficient, *Beta* standardized coefficient

**Table 7:** Relationship between serum lipid parameters and demographic characteristics or lifestyle factors in the Han and Maonan populations

Lipid	Risk factor	B	Std.error	Beta	*t*	*P*
Han						
TC	Height	−0.019	0.007	−0.138	2.642	0.009
	Weight	0.012	0.005	0.125	2.379	0.018
	Body mass index	−0.161	0.055	−0.546	−2.950	0.003
TG	Cigarette smoking	−0.026	0.009	−0.190	−3.030	0.003
	Alcohol consumption	0.013	0.005	0.139	2.652	0.008
	Waist circumference	0.041	0.017	0.227	2.590	0.013
HDL-C	Alcohol consumption	0.002	0.001	0.132	2.516	0.012
LDL-C	Cigarette smoking	−0.022	0.008	−0.194	−3.049	0.005
	Waist circumference	0.017	0.006	0.157	2.998	0.003
	Glucose	0.040	0.017	0.073	2.290	0.022
ApoA1	Alcohol consumption	0.002	0.000	−0.290	5.228	0.000
	Cigarette smoking	0.004	0.002	0.150	2.806	0.006
	Age	0.001	0.001	0.082	2175	0.030
	Weight	0.004	0.002	0.132	2.154	0.032
ApoB	Gender	−0.052	0.016	−0.129	−3.331	0.001
	Waist circumference	0.006	0.001	0.218	4.291	0.000
ApoA1/ApoB	Cigarette smoking	0.004	0.002	0.164	2.725	0.007
	Weight	0.050	0.021	0.124	2.018	0.040
	Glucose	−0.026	0.010	−0.080	−2.592	0.010
Maonan						
TC	Weight	0.062	0.024	0.561	2.621	0.009
TG	Alcohol consumption	0.003	0.001	0.144	2.547	0.011
	Height	−0.112	0.050	−0.512	−2.204	0.041
	Waist circumference	0.038	0.017	0.196	2.284	0.023
HDL-C	Gender	0.102	0.051	0.138	2.016	0.045
	Alcohol consumption	0.002	0.001	0.152	2.265	0.024
	Glucose	−0.027	0.008	−0.097	−3.548	0.000
LDL-C	Gender	0.276	0.055	0.169	5.060	0.000
	Cigarette smoking	0.011	0.006	0.103	2.476	0.010
	Alcohol consumption	0.015	0.005	0.193	2.896	0.003
	Height	−0.006	0.003	−0.259	2.237	0.025
	Pulse pressure	−0.016	0.005	−0.208	−3.232	0.001
ApoB	Gender	0.085	0.013	0.214	6.647	0.000
	Cigarette smoking	0.004	0.001	0.149	2.458	0.009
	Alcohol consumption	0.000	0.000	−0.065	−2.026	0.043
ApoA1/ApoB	Alcohol consumption	0.011	0.004	0.196	2.981	0.003
	Age	−0.003	0.001	−0.096	−3.138	0.002
	Waist circumference	−0.017	0.002	−0.312	−6.653	0.000

*TC* total cholesterol, *TG* triglyceride, *HDL-C* high-density lipoprotein cholesterol, *LDL-C* low-density lipoprotein cholesterol, *ApoA1* apolipoprotein A1, *ApoB* apolipoprotein B, *ApoA1/ApoB* the ratio of apolipoprotein A1 to apolipoprotein B; *B* unstandardized coefficient, *Beta* standardized coefficient

## Discussion

In the current study, we revealed for the first time that: (i) the genotypic and allelic frequencies of four *LIPG* SNPs and their haplotype distribution were different between the Maonan and Han nationalities; (ii) we successfully replicated the association between rs2000813, rs3813082 and rs2097055 SNPs and serum HDL-C levels in the both ethnic groups; the association between rs2000813 SNP and serum ApoA1 and TC levels in the Han population; and the association between rs2097055 SNP and serum LDL-C, ApoB, TG levels, and between rs3819166 SNP and serum ApoA1 levels in the Maonan population. (iii) The T-C-T-A, T-T-C-G and G-T-C-G haplotypes were associated with serum HDL-C levels in the Maonan and Han participants; the T-C-T-A haplotype was associated with ApoA1 levels and the T-T-C-G haplotype was correlated with LDL-C levels in the Maonan subjects.

Serum HDL-C concentration is a major determinant of susceptibility to CVD and is also an inverse correlation to the progression of coronary atherosclerosis [37]. In addition, extensive clinical data showed that each increase 1% in serum level of HDL-C can decrease cardiovascular risk by 2–3% [38]. Previous studies have demonstrated that serum HDL-C levels are regulated in part by members of the lipase enzyme family. Two members of this family, HL and LPL are important in the processing of lipids carried within lipoproteins and probably also in the uptake of lipoprotein particles into cells. The *LIPG* is the most recent member assigned to the TG lipase family, which is involved in the metabolism of lipoproteins, especially HDL-C [39] and in monocyte recruitment during the early inflammation step of atherosclerosis [40]. In contrast to HL, *LIPG* has relatively less TG lipase activity and substantially more phospholipase activity and can hydrolyze HDL phospholipids ex vivo [16]. One previous experimental report has demonstrated that high-level overexpression of *LIPG* in the liver by adenovirus-mediated gene transfer results in a significant decrease in HDL-C and ApoA1 [41], whereas antibody inhibition studies in wild-type and *LIPG* knockout mice have also confirmed that inhibition of *LIPG* causes increased HDL-C levels [19, 42]. However, lipid studies in murine models revealed that *LIPG* could be an important physiological regulator of HDL metabolism and motivate further studies of *LIPG* in human ethnics.

Since 2002, Delemos et al. first discovered 17 polymorphic loci of the *LIPG*, however, whether the *LIPG* polymorphism has an impact on lipid levels and CVD pathogenesis remains unclear. The rs2000813 SNP was a missense polymorphism in exon 3 and results in a change at codon 111 of the *LIPG* from threonine to isoleucine [20]. To date, several researches have failed to validate the association between the rs2000813 genetic variation and serum HDL-C level, but other studies found this polymorphism was correlated to HDL-C level and could also reduce the risk of CVD. Ma et al. [42] previously reported that this variant was significantly associated with HDL-C levels in a well-characterized population of 372 individuals from the Lipoprotein and Coronary Atherosclerosis Study. Modest associations of the rare allele of rs2000813 SNP were identified with HDL-C, and ApoA1 levels in 541 adult Japanese Americans [25]. Besides this, Tang et al. [21] carried out a study including 530 Chinese subjects to investigated the association of the common variations and the risk factors of CAD, they showed that the rare allele of rs2000813 SNP significantly reduced the CAD susceptibility. On the contrary, Shimizu et al. [22] found no association between the rs2000813 polymorphism and HDL-C levels in Japanese. Jensen et al. also founded no relationship between the risk of CAD and T allele of rs2000813 SNP in Caucasian populations [23]. Among the common variations in the *LIPG*, the rs3813082 SNP in the promoter region has been associated with serum HDL-C level in diverse ethnic groups [24, 25]. However, the potential association of rs3819166 and rs2097055 SNPs and serum lipid levels has not previously reported in different populations. In the present study, we found that the rare allele carriers of rs3813082, rs2000813 and rs2097055 SNPs in both Maonan and Han populations had higher HDL-C levels than the rare allele non-carriers. The T allele carriers of rs2000813 SNP in the Han participants had higher serum ApoA1 level than the T allele non-carriers. The C allele carriers of rs2097055 SNP in the Maonan subjects had lower TG, LDL-C and ApoB levels than the C allele non-carriers; and the A allele carriers of rs3819166 variation had lower ApoA1 levels than the A allele non-carriers.

The different observations of the association between the four detected SNPs and serum lipid levels may be explained, at least in part, by the different genotype distribution among different races or ethnic groups. It has been noted that the genotype and allele frequencies of rs2000813 SNP were inconsistent among different populations. In a previous study, the T allele frequency of rs2000813 was reported differently in White and Black (31.2 and 10.3%, respectively) [20]. In addition, the T allele frequency was observed to be 29% in Caucasians [42] and 26% in Japanese [43]. Besides this, the genotypic and allelic frequencies of *LIPG* rs2097055, rs3819166 and rs3813032 SNPs in diverse racial/ethnic groups are not well known. According to the International 1000 Genomes data-base (https://www.ncbi.nlm.nih.gov/variation/tools/1000genomes/), the rs2097055 frequencies of CC, CT genotypes and C allele were 53.57, 21.43 and 46.1% in European; the rs3819166 frequencies of AA, AG genotypes and A allele were 4.00, 31.86 and 20.35% in European; respectively. In the current study, we showed that the allelic frequencies of the four SNPs were distinguished between Maonan and Han ethnic groups. The minor allele frequency of the rs2000813, rs3813082 and rs2097055 were significantly higher in Han than in Maonan subjects (27.05% *vs.* 23.71%, *P* = 0.007; 21.80% *vs.* 16.99%, *P* = 1.17E-05 and 39.53% *vs.* 35.94%, *P* = 0.009; respectively), but the prevalence of the rs3819166A allele (MAF) was significantly lower in the Han than in the Maonan ethnic groups (43.64% *vs.* 47.34%, *P* = 0.009). Generally, the minor allele or rare homozygote genotype frequencies of the 4 detected SNPs in Maonan or Han ethnic groups were significant different from European ancestries. These results suggest that the prevalence of the minor allelic or rare homozygote genotypic frequencies and their haplotypes of the four *LIPG* SNPs might have a racial/ethnic-specificity.

Another reason might be attributed to the differences in LD pattern among the study ethnic groups. In the present study, we showed that there were significantly different in the T-C-T-A, T-C-T-G, T-T-C-G and G-T-C-G haplotype frequencies between the Maonan and Han populations. Moreover, we also found that the T-C-T-A haplotype carriers had lower serum HDL-C concentration; the T-T-C-G and G-T-C-G haplotype carriers had higher serum HDL-C level than the haplotype non-carriers in the two ethnic groups. The haplotypes which combined four SNPs might be explain much more serum lipid variation than any single SNP alone, especially for HDL-C. Above all, ethnic differences in the LD pattern could partially account for the discrepancy in the association of these selected SNPs with serum lipid levels among diverse populations.

Although dyslipidemia is strongly associated with a genetic component, the demographic characteristics, dietary habits and lifestyle factors have been shown to reinforce lipid profile disorders. Reduction in saturated fatty acid (SFA) consumption is traditionally a major focus of dietary recommendations to reduce dyslipidemia and CAD risk [44]. Previous meta-analysis revealed that every 1% alteration in total energy from SFA will lead to a change in TG of l.9 mg/dl; LDL-C of 1.8 mg/dl and HDL-C of 0.3 mg/dl [45]. The Maonan people have custom to eat pickle sour meat, snails and animal offals which contain abundant SFA. This preference of high-fat diet may give rise to higher blood pressure, serum TG and lower ApoA1 levels in Maonan than in Han ethnic groups. In the present study, we also found that the Maonan population had higher the percentage of subjects who consumed alcohol than the Han population (*P* < 0.01). The influences of alcohol drinking on the levels of lipid exhibit both positive and negative results. The effects of medium alcohol consumption on lipid metabolism, especially the increase of serum HDL-C and ApoA1 concentrations, are thought to greatly contribute to the cardio-protective action of alcohol [46]. On the contrary, excessive intake of alcohol has been proved to cause hypertriglyceridemia [47]. For example, Perissinotto et al have indentified that 30 g of alcohol daily was associated with a plasma TG increase of 5.69 mg/dl [48]. Therefore, the results of exposure to different lifestyle and environmental factors probably further modify the association of genetic variations and serum lipid levels in our study populations.

### Limitations

There are several potential limitations in our study. First, the sample size was relatively small compared to many GWASes and replication studies. Thus, further studies with larger sample sizes are needed to confirm our results. Second, we were not able to alleviate the effect of diet and several environmental factors during the statistical analysis. Third, although we have detected the association of several *LIPG* SNPs and serum lipid levels, there are still lots of lipid-related SNPs and the interactions of SNP-SNP and/or SNP-environmental factors. Thus, the association of *LIPG* SNPs and serum lipid levels still needed to verified in different ethnic groups, and the gene expression in adipose tissue should be detected in further studies.

## Conclusions

The present study shows that the genotypic frequencies of the rs3813082 SNP and the allelic frequencies of the four SNPs (rs3813082, rs2000813, rs2097055 and rs381966) were different between the two ethnic groups. The SNPs of rs3813082, rs2000813 and rs2097055 were associated with HDL-C in the Han and Maonan ethnic groups. The rs2000813 SNP was associated with serum TC and ApoA1 levels in Han nationality. On the other hand, the levels of LDL-C, ApoB and TG were correlated to the rs2097055 SNP, and ApoA1 was associated with the rs3819166 polymorphism in Maonan minority. The frequencies of haplotypes among the 4 SNPs were also different between the Han and Maonan populations and the commonest haplotype was rs3813082T-rs2000813C-rs2097055T-rs3819166A. The *LIPG* polymorphisms and their haplotypes (T-C-T-A, T-T-C-G and G-T-C-G) were associated with serum lipid traits. These results suggest that the differences in lipid phenotypic variations between the two populations might partially attribute to the *LIPG* mutations, their haplotypes and G × E interactions.

## Abbreviations

ANCOVA: Analysis of covariance
Apo: Apolipoprotein
BMI: Body mass index
CAD: Coronary artery disease
CVD: Cardiovascular disease
DNA: Deoxyribonucleic acid
DVT: Deep venous thrombosis
G × E: Gene-environment
GWAS: Genome-wide association study
HDL-C: High-density lipoprotein cholesterol
HL: Hepatic lipase
LD: Linkage disequilibrium
LDL-C: Low-density lipoprotein cholesterol
*LIPG*: Endothelial lipase gene
LIPH: Lipase member H
LPL: Lipoprotein lipase
MAF: Minor allele frequency
PCR: Polymerase chain reaction
PNLIP: Pancreatic lipase
RFLP: Restriction fragment length polymorphism
SFA: saturated fatty acid
SNP: Single nucleotide polymorphism
TC: Total cholesterol
TG: Triglyceride
